# Cost of childhood RSV management and cost-effectiveness of RSV interventions: a systematic review from a low- and middle-income country perspective

**DOI:** 10.1186/s12916-023-02792-z

**Published:** 2023-03-31

**Authors:** Rachel Wittenauer, Clint Pecenka, Ranju Baral

**Affiliations:** 1 Comparative Health Outcomes, Policy, and Economics (CHOICE) Institute, University of Washington, Seattle, WA USA; 2 PATH, Center for Vaccine Innovation and Access, Seattle, WA USA

**Keywords:** Respiratory syncytial virus, Cost-of-illness, Cost-effectiveness, Low- and middle-income countries, Systematic review

## Abstract

**Background:**

Approximately 97% of global deaths due to RSV occur in low- and middle-income countries (LMICs). Until recently, the only licensed preventive intervention has been a shortacting monoclonal antibody (mAb), palivizumab (PVZ) that is expensive and intensive to administer, making it poorly suited for low-resource settings. Currently, new longer acting RSV mAbs and maternal vaccines are emerging from late-stage clinical development with promising clinical effectiveness. However, evidence of economic value and affordability must also be considered if these interventions are to be globally accessible. This systematic review’s objective was to summarise existing evidence on the cost-of-illness (COI) and cost-effectiveness of RSV prevention interventions in LMICs.

**Methods:**

We conducted a systematic literature review using the Embase, MEDLINE, and Global Index Medicus databases for publications between Jan 2000 and Jan 2022. Two categories of studies in LMICs were targeted: cost-of-illness (COI) of RSV episodes and cost-effectiveness analyses (CEA) of RSV preventive interventions including maternal vaccines and long-acting mAbs. Of the 491 articles reviewed, 19 met the inclusion criteria.

**Results:**

COI estimates varied widely: for severe RSV, the cost per episode ranged from $92 to $4114. CEA results also varied—e.g. evaluations of long-acting mAbs found ICERs from $462/DALY averted to $2971/DALY averted. Study assumptions of input parameters varied substantially and their results often had wide confidence intervals.

**Conclusions:**

RSV represents a substantial disease burden; however, evidence of economic burden is limited. Knowledge gaps remain regarding the economic value of new technologies specifically in LMICs. Further research is needed to understand the economic burden of childhood RSV in LMICs and reduce uncertainty about the relative value of anticipated RSV prevention interventions. Most CEA studies evaluated palivizumab with fewer analyses of interventions in development that may be more accessible for LMICs.

**Supplementary Information:**

The online version contains supplementary material available at 10.1186/s12916-023-02792-z.

## Background

Respiratory syncytial virus (RSV) causes substantial illness and death globally [1, 2]. It is the second-leading cause of death due to lower respiratory tract infection (LRTI) for all ages and the leading cause of LRTI deaths in children under five years of age [3–5]. The majority of RSV cases and deaths occur in low- and middle-income countries (LMICs), which account for 97% of the total 101,400 RSV-associated deaths in children under 5 years in 2019 [2]. Globally, about 39% of hospital admissions due to RSV occur in children under 6 months of age [2]. Infants of that age have yet to develop fully mature immune systems and traditional vaccination takes time, often requiring multiple immunisations to achieve protection [6]. Furthermore, immunising young infants while naturally transferred RSV-specific maternal antibody is still circulating has the potential to blunt the infant’s response to active vaccination. All these factors would make preventing RSV illness using a traditional vaccine approach difficult to implement in this at-risk population.

Currently, there are no vaccines licensed for RSV and the only widely available preventive product for RSV in young children is palivizumab, a short-acting monoclonal antibody (mAb) product used for passive immunisation against RSV in very high-risk infants and young children. Palivizumab has an estimated efficacy of 55% for the reduction of hospitalisation due to RSV infection [7]. While effective, its high cost and monthly dosing schedule make implementation prohibitive in most low-resource settings [8]. To address these challenges and deploy RSV prevention interventions with fewer implementation barriers, several vaccine and prophylactic products are currently being developed. As of September 2021 (the most recent pipeline summary available), there are 23 vaccine and antibody products for RSV prevention undergoing clinical trials (of which 6 have target indications for only elderly populations at risk), with 17 additional products in preclinical development [9]. One product strategy is maternal immunisation (MI), during which pregnant people are vaccinated against RSV to subsequently pass antibodies along to their newborns for protection during the first several months of life. In November 2021, an MI vaccine showed promising results after a phase 2b study, with potentially 85% efficacy in protecting infants from LRTI due to RSV [10, 11]. Additionally in November 2021, nirsevimab, a single-dose long-acting monoclonal antibody—the other main strategy for prophylactic RSV products— developed by AstraZeneca and Sanofi demonstrated safety and 75% efficacy at preventing RSV in a Phase 3 clinical trial; filing for regulatory approval is expected in early 2022 [12–15]. These two new products and others in the clinical pipeline have the potential to dramatically lower RSV illness and death globally.

However, evidence on the economic burden of RSV and the potential cost-effectiveness of these treatments in LMICs, where the burden is greatest [4, 16], is lacking: a recent systematic review of cost-of-illness for RSV at the global level found that only 3 out of 44 costing studies were conducted in LMICs, highlighting the income and geographic disparity in the available evidence [17]. We note that throughout this paper, “LMIC” refers to low- and middle-income countries. In instances where this analysis refers to specifically lower-middle-income countries, this term is fully spelled out rather than abbreviated as LMIC.

Economic evidence such as the relative value of RSV interventions is important to inform adoption and implementation recommendations. As promising RSV vaccines and mAbs move through late-stage clinical trials and licensure, economic evidence will need to be considered as ministries of health weigh the value of these interventions against real-world budgetary constraints. Evidence on economic burden of RSV and the relative cost-effectiveness of new interventions to prevent RSV illness and death in specific contexts is necessary to make these determinations.

This review identifies and summarises all available evidence regarding the RSV cost-of-illness and cost-effectiveness of RSV prevention interventions for children under five years of age in LMICs. Cost-of-illness studies quantify the resources expended to treat and manage a health condition and are useful to understand the value of interventions addressing those health problems. Cost-effectiveness studies provide evidence informing how resources should be allocated. This will provide insight for (1) the research community on where evidence gaps currently exist; (2) country-level decision-makers in LMICs on the economic burden of RSV and the relative value of RSV interventions specific to their contexts; and (3) donors and global policymakers on RSV-related investment priorities.

## Methods

We conducted a systematic literature review to identify relevant studies for inclusion by following the guidance in the PRISMA guidelines for conduct and reporting of systematic reviews [18, 19]. Our search identified research studies that measured RSV cost-of-illness in children in LMICs and studies that measured the cost-effectiveness of RSV prevention interventions in children in LMICs. We searched the MEDLINE, Embase, and Global Index Medicus publication databases on January 20, 2022, for studies conducted since January 2000. Our search terms included both the specific keyword and the exploded keyword concept in Emtree, when available. These terms included keywords for respiratory syncytial virus, cost and cost-effectiveness, and low- and middle-income countries. The full keywords and limits used in the search are summarised in Additional File 1. The list of low- and middle-income countries was defined using the 2021 World Bank classifications of countries based on gross national income per capita including low-income countries, lower-middle-income countries, and upper-middle-income countries [20].

Characteristics of the literature identified through this search were exported to Microsoft Excel for review. Two authors (RW and RB) reviewed all titles and abstracts to identify studies that merited full article review. After reviewing all relevant full-text articles for relevance, the resulting list of articles was evaluated for quality using the Drummond checklist (see Additional File 2) and for biases reported in the article text. Data for these articles were extracted into Microsoft Excel tables by RW and reviewed by RB.

For the cost-effectiveness studies, the primary outcome measure for data extraction was the incremental cost-effectiveness ratio (ICER) of the intervention in dollars per quality-adjusted life years (QALY) gained or disability-adjusted life years (DALY) averted. When available, the confidence interval of the ICER estimates was extracted as well. The assumed intervention cost, intervention efficacy, and intervention duration used in each article were also extracted. To provide a willingness-to-pay (WTP) reference for contextualising ICERs, the 2020 gross domestic product (GDP) per capita in $USD is also included for each country. A WTP threshold of 1 times GDP per capita can be used as a threshold; however, some guidance recommends 3 times GDP per capita as an appropriate threshold, while others dissuade the decision-making community from using GDP per capita as a reference in any capacity, as is the current stance of the World Health Organization [21]. Accordingly, we included GDP per capita data here for reader reference, but did not use these data to make conclusive determinations about intervention cost-effectiveness in each specific country setting for the purposes of this review.

For the cost-of-illness studies, the primary measures of interest for data extraction were direct and indirect costs and associated confidence intervals when available. When more granular data for specific cost components such as medications, staff costs, and supplies were provided, these were extracted as well. Severity of RSV was classified into outpatient (least severe), ward hospitalisation, and ICU hospitalisation (most severe) based on the setting where each study was conducted. The average length of hospital stay observed in each study was also documented for comparison purposes.

For all studies, all cost estimates were converted to 2020 US dollars by first starting with the local currency used in each study and then adjusting for inflation using country-specific inflation rates before converting back to 2020 US dollars, as recommended by Turner et al.’s adjustment method number 2 [22]. Inflation rates and currency conversion for each year and country were sourced from The World Bank.

The completed PRISMA checklist for this systematic review is included as Additional File 3.

## Results

### Study selection

**Fig. 1:**
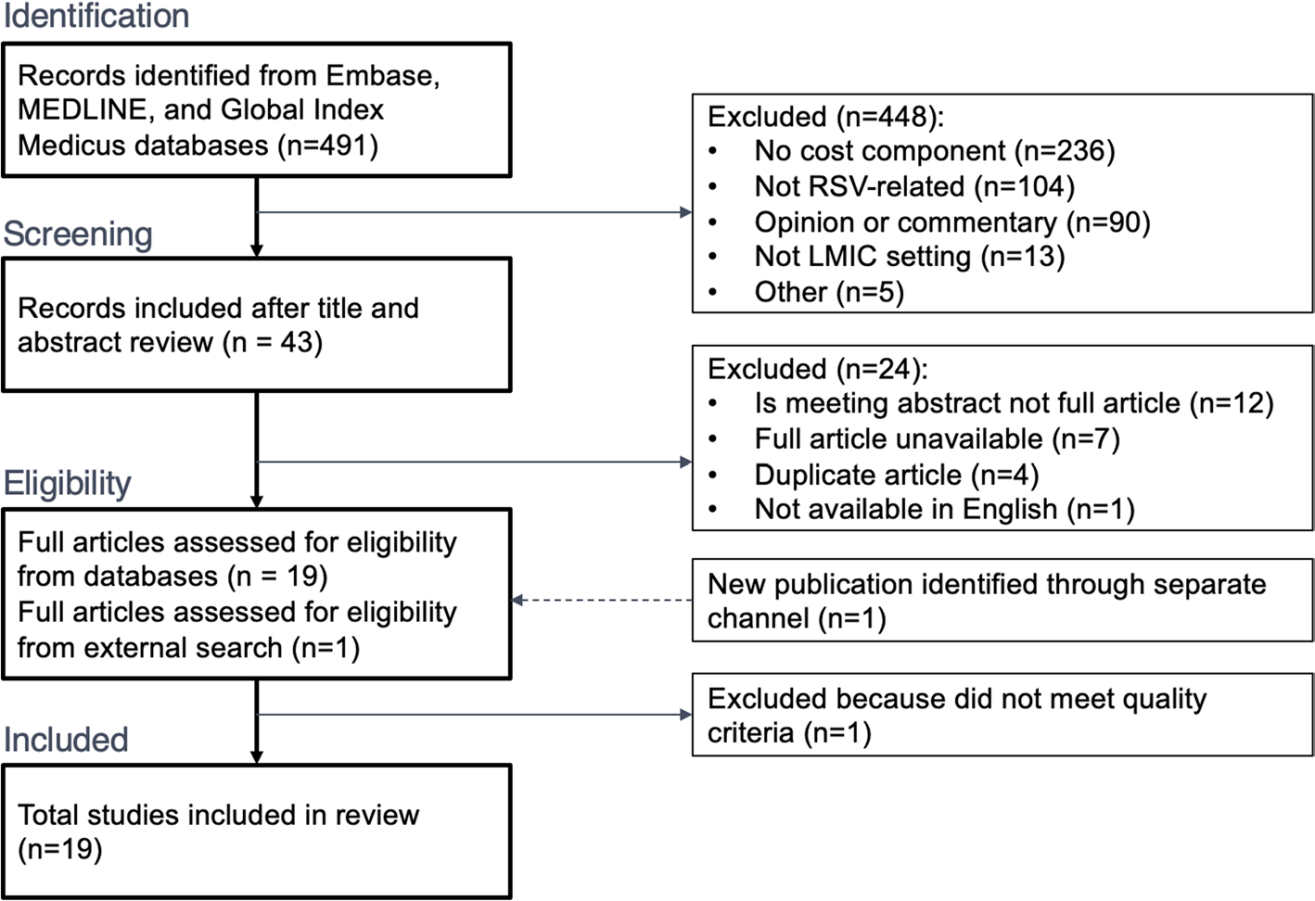
PRISMA flow diagram of included studies. Abbreviations: LMIC, low- and middle-income countries; RSV, respiratory syncytial virus

The initial search terms produced 491 articles for review. Titles and abstracts of all articles were reviewed by RW and RB for possible inclusion, which eliminated 448 studies that did not have a cost component (*n* = 236), were not RSV-related (*n* = 104), were an opinion or commentary (*n* = 90), did not take place in an LMIC setting (*n* = 13), or other reason (*n* = 5). The full text of the remaining 43 articles was then reviewed, and 24 articles were further excluded due to referring to a meeting abstract rather than a full study (*n* = 12), the full text being unavailable (*n* = 7), being duplicate articles (*n* = 4), and not being in English and therefore not adequately interpretable by the study team (*n* = 1). One additional article (Liu et al. [23]) met the keyword criteria but was identified via a separate search, as it was still pending full indexing in the Embase database at the time of data extraction. We then evaluated all remaining 20 articles for data quality using the Drummond checklist, where an article needed to meet > 50% of the available points to be included in this review. Articles that were cost-effectiveness analyses were evaluated using the complete Drummond checklist, while cost-of-illness articles were evaluated against a subset of relevant criteria from the Drummond checklist (see Additional File 2). One article was excluded based on a low score for study quality, leaving 19 total articles for inclusion in this review. No articles were excluded based on the risk of study biases or conflicts of interest. Of these 19 included studies, only 6 have been included in previously-conducted systematic reviews on economics of RSV [6, 17, 24]. This process is summarised in Fig. 1 below.

### Characteristics of Included Studies

The 19 included studies were reviewed and analysed in two groups based on the objective of the study: cost-effectiveness analysis (CEA) of an RSV intervention (*n* = 10) [5, 25–32] or cost-of-illness (COI) estimation of RSV (*n* = 10) [23, 25, 32–40]. One study, Chan et al., included both a CEA and COI component and so are included in both categories of results presented here.

The CEA articles evaluated a variety of interventions, with most studies evaluating more than one intervention: short-acting mAb (palivizumab) (*n* = 6), maternal immunisation (*n* = 5), long-acting mAb (*n* = 4), and paediatric immunisation (*n* = 2). Seven of the ten CEAs took place in upper-middle-income countries (UMIC), two in lower-middle-income countries, and one in a low-income country (LIC). The characteristics of the included CEA studies are summarised in Table 1.

**Table 1:** Cost-effectiveness studies included in the review

Income group^a^	Setting	Author/year	Intervention(s) evaluated	Study objective	Key assumptions	Key measures and results
UMIC	Argentina (ARG)	Farina et al. (2002) [26]	Short-acting mAb palivizumab (PVZ)	To evaluate the cost-effectiveness of PVZ given to premature infants (< 24 months old) with bronchopulmonary dysplasia (BPD) born in a hospital at < 35 weeks and < 28 weeks	Intervention cost: $656 Intervention efficacy: 55% Duration of protection: not reported (NR) Approach: Retrospective cohort study	Outcome measure: Incremental cost-effectiveness ratio (ICER) in USD ($)/hospitalisation averted Results: $15,538
UMIC	Argentina (ARG)	Rodriguez et al., (2008) [41]	PVZ	To perform a cost-effectiveness analysis on PVZ administration for the prevention of RSV-related hospitalisation rate in premature infants (< 24 months old) with and without BPD and with and without siblings in a hospital in Argentina	Intervention cost: $792 Intervention efficacy: 55% Duration of protection: NR Approach: Retrospective cohort study	Outcome measure: ICER $/hospitalisation averted Result: $10,456 among those without BPD and with siblings; $16,837 among those with BPD and with siblings; $34,088 among those with BPD and without siblings; $71,226 among those without BPD and without siblings
UMIC	China (CHN)	Liu et al., (2021) [23]	Maternal immunisation (MI), long-acting mAb, paediatric immunisation (PI)	To evaluate the threshold strategy cost (TSC) of RSV interventions (the maximum cost per child for an intervention to be cost-effective)	Intervention cost: NR Intervention efficacy: MI 71.6%; mAb 100%; PI 100% waning to 70% by year 5 Duration of protection: MI 4 months; mAb time-varying; PI time-varying Approach: Static cohort model	Outcome measure: TSC Result: Under willingness-to-pay of 1 GDP per capita per QALY gained, the TSC of RSV intervention ranged between $2.40 and $14.70 for MI; $19.90 and $144.20 for mAb; $28.70 and $201 for PI
UMIC	Mexico (MEX)	Salinas-Escudero et al., (2012) [29]	PVZ	Evaluated the cost-effectiveness of PVZ prophylaxis for the reduction of RSV-associated complications in preterm patients (< 32 weeks and < 29 weeks) during RSV season in Mexico	Intervention cost: $661 Intervention efficacy: 55% Duration of protection: NR Approach: Decision tree model	Outcome measure: ICER $/QALY Result: $17,532 among preterm births < 29 weeks and $20,760 among preterm born 29–32 weeks
UMIC	Turkey (TUR)	Oncel et al., (2012) [30]	PVZ	Evaluate the cost-effectiveness of RSV prophylaxis with PVZ in infants (< 24 months) from two hospitals in Turkey	Intervention cost: $516 Intervention efficacy: NR Duration of protection: NR Approach: Retrospective cohort study	Outcome measure: Average (mean) cost of treatment Result: $5019 for those treated with PVZ versus $1934 for those untreated
UMIC	Turkey (TUR)	Pouwels et al., (2016) [31]	MI, PI	To evaluate the potential cost-effectiveness of RSV vaccination in infants and/or pregnant women in Turkey	Intervention cost: MI $13; PI $13 Intervention efficacy: MI 60%; PI 60% Duration of protection: MI 5 months; PI 2 years Approach: Multi-cohort static Markov model	Outcome measure: ICER $/QALY Result: MI TL 60,638; PI TL 51,969
UMIC	Malaysia (MYS)	Chan et al., (2003) [32]	PVZ	Determine the direct cost of resource utilisation in the treatment of children hospitalised with RSV chest infection and the potential cost savings with passive immunisation for high-risk infants	Intervention cost: $3360 Intervention efficacy: 55% Duration of protection: NR Approach: Retrospective cohort study	Outcome measure: ICER $/hospitalisation averted Result: $2921
Lower- middle-income country	Global (Gavi countries)	Li et al., (2020) [5]	MI, long-acting mAb	Use a country-specific model to evaluate the key drivers of the cost-effectiveness of maternal vaccination and infant mAb for 72 Gavi countries	Intervention cost: MI $3; mAb $6 Intervention efficacy: MI 75%; mAb 60% Duration of protection: MI 5 months; mAb 6 months Approach: Multi-country static cohort model	Outcome measure: ICER $/DALY Result: MI $1893; mAb $2769
Low- and middle-income countries	Global (low- and middle-income countries)	Baral et al., (2021) [28]	MI, long-acting mAb	Use a country-specific model, evaluate the potential impact and cost-effectiveness of two interventions to prevent childhood RSV—a maternal vaccine and a mAb	Intervention cost: MI $3.22; mAb $3.22 Intervention efficacy: MI 42%; mAb 70% Duration of protection: MI 6 months; mAb 6 months Approach: Static cohort model	Outcome measure: ICER $/DALY Result: MI: $1342; mAb: $431
LIC	Mali (MLI)	Laufer et al., (2021) [27]	PVZ, MI, long-acting mAb	Model the estimated likelihood of RSV preventive interventions being cost-effective in Mali	Intervention cost: PVZ NR; MI $1.38; mAb $1.38 Intervention efficacy: PVZ 55%; MI 56%; mAb 70% Duration of protection: PVZ 1mo; MI 3 months; mAb 5 months Approach: Decision tree model	Outcome measure: ICER $/DALY Result: PVZ $4280; MI $8020; mAb $1656

Notes: *BPD* bronchopulmonary dysplasia, *DALY* disability-adjusted life year, *ICER* incremental cost-effectiveness ratio, *LIC* low-income country, *mAb* monoclonal antibody, *MI* maternal immunisation, *NR* not reported, *PI* paediatric immunisation, *PVZ* Palivizumab a short-acting monoclonal antibody, *QALY* quality-adjusted life year, *TL* Turkish Lira, *TSC* threshold strategy cost, *UMIC* upper-middle-income country

Results in this table are reported in the year and currency of the original article

^a^Country income group is sourced from 2021 World Bank country classifications by gross national income per capita

The COI studies most often evaluated the cost of RSV from the payer’s perspective. Results from the included COI studies are grouped by RSV severity: outpatient care (*n* = 2), mild hospitalised ward care (*n* = 4), and severe or intensive care hospitalisation (*n* = 9). Eight of the ten COI studies took place in UMICs, one in a lower-middle-income country, and one in a LIC. The characteristics of the included COI studies are summarised in Table 2.

**Table 2:** Cost-of-illness studies included in the review

Income group^a^	Setting	Author	Study objective, sample size (*n*), and population	Key measures and results
UMIC	Argentina (ARG)	Marcone et al., (2015) [34]	Objective: Estimate the cost of hospitalisations due to aetiology-specific ALRI among children aged ≤ 5 years and costs incurred during their hospitalisations (*n* = 358) Subgroups analysed: Inpatient only Approach: Prospective cohort design, administrative hospital records	Outcome measure: Direct medical costs Length of stay: 3 days (IQR: 2–4) Results: $529 per hospitalisation episode
UMIC	China (CHN)	Zhang et al., (2014) [36]	Objective: Estimate the direct medical cost of laboratory-proven RSV children hospitalised in Suzhou, China (*n* = 2721) Subgroups analysed: Ward and ICU Approach: Retrospective review of medical records	Outcome measure: Direct medical costs Length of stay: 8 days (IQR: 7–9) Results: $565.36 per episode for children seen in ward; $909.65 per episode per children admitted to ICU
UMIC	Colombia (COL)	Rodriguez-Martinez (2020) [25]	Objective: Evaluate the direct medical costs associated with bronchiolitis hospitalisations caused by infection with RSV among children aged < 2 years in Bogota, Colombia (*n* = 89) Subgroups analysed: Ward and ICU Approach: Retrospective review of medical records	Outcome measure: Direct medical costs Length of stay: 7 days (+ / − sd 4.4 days) Results: US$518.0 per episode per children seen in ward; $2749.7 per episode per children admitted to ICU
UMIC	Colombia (COL)	Buendia et al., (2021) [37]	Objective: Evaluate the medical costs associated with bronchiolitis hospitalisations caused by RSV infection among infants aged < 2 years in Colombia (*n* = 193) Subgroups analysed: Inpatient only Approach: Retrospective review of medical invoice and health records	Outcome measure: Direct and indirect medical costs Length of stay: 5.88 days Results: Cost per episode for children in ICU $580 (direct); $101 (indirect)
UMIC	Malaysia (MYS)	Chan et al., (2003) [32]	Objective: Determine the direct cost of resource utilisation in the treatment of children hospitalised with RSV chest infection and the potential cost savings with passive immunisation for high-risk infants (*n* = 216) Subgroups analysed: Full-term and pre-term children Approach: Retrospective review of medical records and resource use data	Outcome measure: Direct medical costs Length of stay: 3.9 days for full-term, 9.0 days for pre-term Results: Direct cost per episode among full-term children: $216; and among pre-term: $2485
UMIC	Malaysia (MYS)	Sam et al., (2021) [39]	Objective: Determine the socioeconomic burden of children < 5 years hospitalised with ARI by interviewing patients’ carers and obtaining unsubsidised hospital costs (*n* = 74) Subgroups analysed: Inpatient only Approach: Prospective study	Outcome measure: Direct medical, non-medical costs, and indirect costs Length of stay: 4 days (IQR: 3–5) Results: Direct medical cost per episode $756; non-medical costs $31; indirect costs $98
UMIC	Mexico (MEX)	Comas-Garcia et al., (2020) [40]	Objective: ﻿Assess the impact and cost of nosocomial RSV infections in a NICU (*n* = 24 RSV, 24 control) Subgroups analysed: Inpatient only Approach: Retrospective cohort study	Outcome measure: Direct medical costs Length of stay: 24 days (vs 13 days for control of hospitalised with no RSV infection) Results: Direct medical cost of nosocomial RSV infection among children admitted in NICU $6922 per episode
UMIC	Thailand (THA)	Bhuket et al., (2002) [33]	Objective: Determine the economic burden in the management of ALRI from the patient’s perspective at Takhli District. Hospital (*n* = 165) Subgroups analysed: Outpatient and inpatient Approach: Cross-sectional interviews and records review	Outcome measure: Direct medical, direct non-medical, and indirect medical costs Length of stay: NR Results: Cost per episode for outpatient $312 (direct), $118 (non-medical), $73 (indirect); cost per episode for inpatient $1134 (direct), $387 (non-medical), $222 (indirect)
Lower-middle-income country	Bangladesh (BGD)	Bhuiyan et al., (2017) [35]	Objective: Estimate the costs of severe RSV illness requiring hospitalisation among children < 5 years and the associated financial impact on households in Bangladesh (*n* = 39) Subgroups analysed: inpatient only Approach: Cross-sectional interviews	Outcome measure: Direct and indirect medical costs, household costs Length of stay: 5 days (IQR: 3–6) Results: Total cost per episode of RSV $94; direct cost per episode $62; indirect cost per episode $19; household out-of-pocket costs $143 (24% of household monthly income)
LIC	Malawi (MWI)	Baral et al. (2020) [38]	Objective: Estimate the household and health system costs of managing RSV and other respiratory pathogens in Malawian infants (*n* = 426) Subgroups analysed: outpatient and inpatient Approach: Cross-sectional interviews and medical record reviews	Outcome measure: Direct and indirect medical costs, household costs Length of stay: 3 days (IQR: 2–4) Results: Total cost per episode of RSV $62.26 (inpatient) and $12.51 (outpatient); household costs accounted for 20% of cost per episode which for low-income families was 32% of monthly household income

Notes: *ARI* acute respiratory infection, *ICU* intensive care unit, *IQR* interquartile range, *LIC* low-income country, *NR* not reported, *RSV* respiratory syncytial virus, *UMIC* upper-middle-income country. Results in this table are reported in the year and currency of the original article

^a^Country income group is sourced from the 2021 World Bank country classifications by gross national income per capita

### Findings from included studies

Broadly, a review of these CEA and COI studies indicates wide-ranging estimates for the cost-effectiveness of specific interventions and estimated cost-of-illness of RSV episodes.

#### Cost-effectiveness

The evaluation results varied widely for each intervention evaluated, and studies evaluated the interventions using different outcome measures. Of the six studies evaluating palivizumab, the ICERs were estimated from $4671/DALY in Mali [27], to $22,863/QALY in Mexico [29]. The ICERs in dollars per hospitalisation averted ranged from $4140 in Malaysia [32] to $71,226 in Argentina [25]. One study reported an average cost per hospitalisation, which was found to be $2416 in those treated with short-acting mAb vs $931 in those untreated [30].

Of the studies evaluating maternal immunisation (MI), ICERs per DALY averted averaged at about $1440 across all LMICs in the study [28]. A study focusing on current and former Gavi-funding-eligible countries as of 2020 [42] reported the average ICER per DALY averted at $2031 [5]. Another study, using country-specific input values in a LIC (Mali), found the cost per DALY averted for a maternal vaccine to be $8753 [27]. One study reported the estimated ICER per QALY gained for an MI product in Turkey to be $118,845/QALY gained [31]. Another recent study from China presented the CEA results as threshold strategy costs (TSCs) based on a willingness-to-pay threshold of 1 GDP per capita. The TSC for MI in this study was found to range between $2.40 and $14.70 across sub-regions [23].

The four evaluations of long-acting mAbs found ICERs of $1687/DALY (Mali) [27], $2971/DALY (Gavi countries) [5], and $462/DALY (LMICs) [28]. The study, which reported results by TSC rather than ICER, found that the TSC for mAbs ranged between $19.90 and $144.20 depending on the region (China) [23].

Finally, two studies evaluated the cost-effectiveness of a hypothetical paediatric immunisation program (PI). One study found the ICER was $101,854/QALY gained (Turkey) [31]. The second study reported results by TSC rather than ICER and found that the TSC for PI ranged between $28.70 and $201.00 depending on the region (China) [23].

**Fig. 2:**
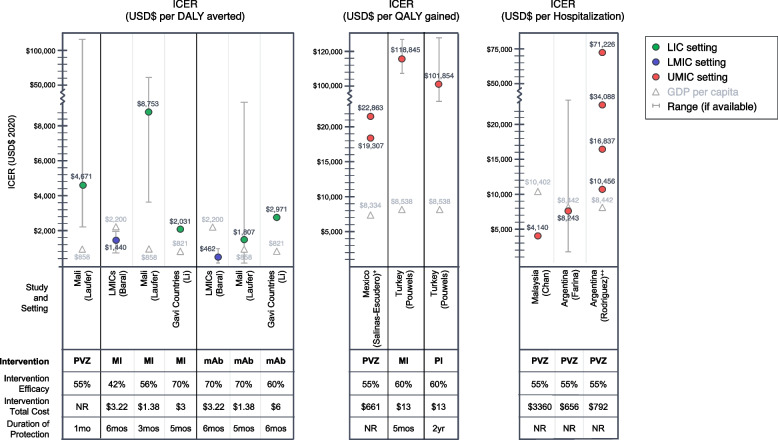
Findings from cost-effectiveness studies. Notes: ^+^ Study reports results in two subgroups: infants born at < 29 weeks ($19,307) vs. born 29–32 weeks (22,863); ^++^ Study reports results in four subgroups: those without BPD and with siblings ($10,456), those with BPD and with siblings ($16,837), those with BPD and without siblings ($34,088), and those without BPD and without siblings ($71,226). Currency is reported in 2020 USD. Abbreviations: BPD, bronchopulmonary dysplasia; DALY, disability-adjusted life year; GDP, gross domestic product; ICER, incremental cost-effectiveness ratio; LIC, low-income country; LMIC, low- and middle-income countries; mAb, Long-acting monoclonal antibody; MI, maternal immunisation; NR, not reported; PI, paediatric immunisation; PVZ, pavilizumab; QALY, quality-adjusted life year; UMIC, upper-middle-income country; USD, United States dollars

These estimates often had wide confidence intervals and sensitivity analyses which indicated a wide range of possible ICERs depending on characteristics including intervention efficacy and price. As seen in Fig. 2, the assumed intervention efficacy, intervention cost, and duration of protection were different among all studies, except for palivizumab, which has a more clearly demonstrated real-world effectiveness than the other intervention types.

Figure 2 illustrates the ICER result of each study by the outcome measure and which intervention was evaluated. For comparison, the figure also includes the GDP per capita of each study setting, which can be considered a reference for a threshold in determining cost-effectiveness [43]. CEA estimates across all outcome measures are higher than the GDP per capita threshold in almost all cases. Applying a WTP threshold of 1 × GDP per capita per DALY averted (to the ICER estimates in DALYs only), nearly all study results are deemed to not be cost-effective. The exception is the study by Baral et al. (2021) whose modelling approach found that overall in LMICs, both maternal immunisation and long-acting mAb would be cost-effective [28]. However, applying other WTP thresholds such as 3 × GDP per capita could lead to a higher number of these included studies concluding that the evaluated interventions are cost-effective.

#### Cost-of-illness

The estimated cost-of-illness for RSV also varied widely. Five of the studies estimated both direct and indirect costs, one study also estimated direct non-medical costs, and all ten estimated direct medical costs per episode. Direct costs typically included room charges, medications, consumables, diagnostics, and consult services. Direct non-medical costs included costs incurred by households in transportation and subsistence, and indirect costs included lost productivity in wages of the child’s caretaker during the RSV episode.

**Fig. 3:**
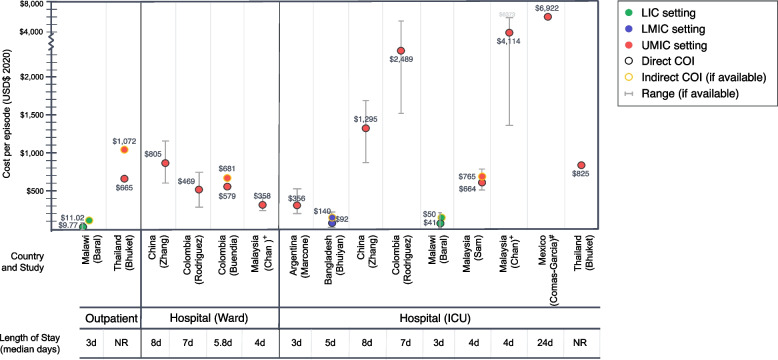
Findings from cost-of-illness studies. Notes: + Comparison groups are for full-term infants vs. pre-term infants, which are included in the Ward and ICU categories, respectively, for comparison by disease severity; # Study evaluates the cost of nosocomial RSV infections. Currency is reported in 2020 USD. Abbreviations: COI, cost-of-illness; ICU, intensive care unit; LIC, low-income country; LMIC, low- and middle-income country; NR, not reported; UMIC, upper-middle-income country; USD, United States dollars

Among studies measuring outpatient care, cost estimates for direct costs per illness episode were $9.77 (Malawi) [38] and $665 (Thailand) [33] and corresponding estimates for indirect costs were $11.02 (Malawi) and $1074 (Thailand). For mild RSV cases or cases seen in the health facility ward, direct costs per episode were $805 (China) [36], $469 (Colombia) [25], $579 (Colombia) [37], and $358 (Malaysia) [32]. Indirect costs per episode were reported to be $681 (Colombia) [37]. For severe RSV cases in the intensive care unit cost per episode ranged from $92 (Malawi) [38] to $4,114 (Malaysia) [32]. One study in Mexico estimated cost-per-illness episodes to be $6922 [40], however, it should be noted these estimates were specifically for nosocomial RSV infection which may have different cost characteristics than the other studies, which evaluated community-acquired RSV cases. Another study in Malaysia [32] compared the cost of care among full-term infants vs. pre-term infants, which are included in the “ward” and “ICU” categories, respectively, for comparison by disease severity. These estimates are summarised in Fig. 3.

Eight of the ten COI studies provided further granular data on components of the direct medical costs. Direct cost per hospitalisation was driven by a variety of cost components, with the largest drivers often being room charges, diagnostics, and medications. In the study evaluating outpatient RSV, the largest cost component was diagnostics. In the four studies evaluating mild, ward-attended RSV, the largest cost drivers were room charges [25, 37], medications [36], and diagnostics [32]. The main driver of ICU-attended RSV illness costs was room charges [25, 34, 39], followed by medications [36], diagnostics [38], consults [40], and others [32]. Sub-components of direct costs were not available for Bhuket et al. [33] and Bhuiyan et al. [35]. These data are summarised in Additional File 4.

## Discussion

Among the cost-effectiveness analyses, there was a wide range of estimated ICERs. Generally, the ICERs for hypothetical MI or long-acting mAb are lower (more favourable) than for analyses of the existing short-acting mAb product palivizumab. However, when comparing ICERs to the threshold of 1 GDP per capita in each study setting, it is unclear whether MI or long-acting mAbs will be broadly cost-effective. Cost-effectiveness analyses to date rely heavily on efficacy assumptions and implementation setting; as such the available estimates and sensitivity analyses encompass a wide range of possible conclusions about the cost-effectiveness of RSV interventions. As more precise data on intervention efficacy and duration of protection become available from ongoing clinical trials for new long-acting mAb and maternal vaccine products, it would allow more accurate inputs for ICER estimation and relative value conclusions in future CEAs.

There were several gaps identified in the available CEA evidence. Only three studies were conducted in low-income or lower-middle-income country settings, while the rest were in upper-middle-income countries. More evidence is needed to inform implementation decisions on future adoption of RSV prevention products in these countries. Studies from HICs may still be informative for decision-making as components of a larger package of evidence but are not sufficient. Additionally, CEAs were primarily conducted to evaluate palivizumab, with only four studies evaluating hypothetical administration of future vaccines or long-acting mAb products. The identified CEAs primarily quantify direct medical costs and near-term health effects. Of note, clinical trials have shown RSV products to offer protection against non-RSV LRTI [44]. These potential additional benefits, which are not incorporated in current cost-effectiveness analyses, would imply the value of these products is currently under-estimated in preventing the LRTI disease burden. Furthermore, RSV has distinct seasonality in many countries, and provision of RSV interventions in the seasonal transmission settings likely has an important impact on its cost-effectiveness which is an area for future research in LMICs.

Estimates of cost-of-illness also varied widely across studies. There is a wide range of direct medical costs per episode for RSV—for severe RSV, the estimates ranged from $92 in Malawi (Baral et al.) to $4114 in Malaysia (Chan et al.). Relative to the household income or GDP per capita in each of these study settings, RSV represents a meaningful financial burden for families and medical systems. While only two COI studies were identified that reported cost-of-illness in the context of household costs, they found RSV episodes leading to substantial economic burden: accounting for roughly 24% [35] or 37% [38] of their monthly household income. A recent systematic review of RSV models by Zhang et al. [17], which included studies conducted in high-income countries in addition to LMICs, also concluded that RSV “imposed a substantial economic burden on health systems, governments, and society,” which is in alignment with our findings from this LMIC-specific review. Despite the addition of multiple recent studies to the literature, a large gap in understanding remains regarding the financial consequences of RSV episodes in children in LMICs specifically.

In high-income countries, evidence also points to a substantial cost-of-illness burden. A systematic review by Zhang et al. [17] of the cost of RSV globally found that the mean cost per inpatient for RSV management exceeded the total healthcare expenditure per capita in most OECD (Organisation for Economic Co-operation and Development) countries. Of the 41 articles identified in this review, none were from low-income countries, though it noted that inpatient medical costs were higher in middle-income countries than in high-income countries, perhaps due to different thresholds for hospitalisation or the different economic environments [17]. Costs were heterogeneous between included studies, which all applied varying methodologies and examined different subgroups of patients, further highlighting the need for improved evidence on cost of RSV management even in high-income countries. However, the high cost-of-illness burden is apparent and consistent even among high-income and middle-income countries in this review. Evidence on the cost-effectiveness of RSV interventions in HICs also varies by country setting; however, evidence suggests that, despite its high-cost and moderate efficacy, palivizumab is a cost-effective prevention option in settings with a WTP threshold. A review by Mac et al. [8] found that ICERs ranged from $5188 to $791,000 per QALY, though 90% of the 28 included study results were below a WTP of US$50,000 per QALY. However, that high of a WTP threshold is not acceptable in resource-constrained settings.

### Implications for future studies

The review identified several areas which would benefit from future study. For both categories of studies, CEA and COI, only two were conducted in countries in the lowest income tier, and evidence of intervention efficacy and costs in these specific settings will be needed to inform future product adoptions in those settings. Further, both CEA and COI analyses were often limited to a short time horizon and direct medical costs, excluding broader societal and longer-term costs and benefits. Broader implications of RSV disease burden and its preventive measures have been noted in the literature, for example, the possible linkages between childhood RSV and onset of asthma and recurrent wheeze in later years [44], potential of protective effectiveness of RSV interventions against other ALRI [44], and the reduction in antibiotic prescription and use among children vaccinated against RSV [45]. Further, the societal costs of death due to RSV in young children are not included when only considering the direct medical costs of RSV, and the majority of RSV-associated deaths occur in LMICs [2]. While evidence on treatment seeking specifically for RSV-associated conditions in LMICs is quite limited, some evidence indicates that the proportion of outpatient acute respiratory infection presentation is higher in HICs, perhaps due to different healthcare system access and utilisation behaviours [46], but that RSV hospitalisation rates are similar to those of higher-income countries, indicating the high potential of an RSV vaccine [47]. Additionally, the percentage of children under 5 years of age who sought care for pneumonia symptoms, a close proxy for RSV treatment seeking, varies widely by country income level and region and may be as low as 40% in West and Central Africa [48]. The lack of inclusion of these types of costs, benefits, and LMIC-specific healthcare system characteristics in existing studies implies that the estimates described in this review are underestimates of the total RSV health and economic burden from the patient and societal perspective. Future studies should consider these potential effects to gain a comprehensive understanding of the value of RSV prevention interventions.

### Strengths and limitations

This systematic review offers several strengths. It is the most comprehensive review to date of the economic burden of RSV specific to LMICs: while previous cost-of-illness and cost-effectiveness reviews have included some of the same studies in their findings, the majority of included evidence and perspectives in those reviews was from high-income countries [6, 17]. This review specifically focuses on illuminating the evidence gaps in LMICs, where the RSV burden is greatest [2, 4, 5]. The process of article identification and review followed PRISMA best practices for conduct and reporting of systematic reviews. Two authors independently reviewed the database search results for inclusion in the review, strengthening the reliability and repeatability of this study. This search also used a broad range of keyword terms to capture the economic value and included more than two decades of articles in the scope of the search to bolster the comprehensiveness of the review.

There are also several limitations to this review. First, this review was limited to evaluations specifically conducted on RSV and RSV interventions. It is possible there are studies providing relevant cost-of-illness for hospitalisation with LRTI that, while not specific to RSV, may still be informative estimates that would strengthen the value case for RSV illness prevention. Second, most of the COI studies evaluated cost-per-hospitalisation, and thus do not measure the direct and indirect costs of home-attended mild RSV, which may still have economic and productivity costs for families of mildly ill children. Lastly, search terms were limited to English-only studies, which is not the official language of many LMICs and may have excluded relevant evidence for inclusion in this study.

## Conclusion

Several recent studies have evaluated the economic burden of RSV and the potential economic impact of RSV prevention interventions in low- and middle-income countries. The cost of RSV presents a significant burden in terms of medical costs when taken in the context of local GDP per capita. However, important evidence gaps remain regarding the economic value of new RSV prevention interventions, specifically in low-income settings. In aggregate, evidence to date is uncertain as to whether existing and late-stage-development interventions will be cost-effective in these settings. Additional studies should incorporate the latest disease burden data, intervention characteristics and data specific to LMICs to inform adoption decisions for these new interventions.

## Supplementary Information

**Additional file 1.** Search keywords. This file lists all search terms that were used to search Embase, MEDLINE, and Global Index Medicus via the Embase search interface. The three components of the search terms were groups of terms related to each topic: “RSV”, “Economics”, and “Low and middle income countries”.

**Additional file 2.** Article quality checklists. **Table S3.1.** This table summarizes the quality score of each evaluated cost-effectiveness analysis article for inclusion, using the Drummond checklist. **Table S3.2.** This table summarizes the quality score of each evaluated cost-of-illness article for inclusion, using the Drummond checklist.

**Additional file 3.** PRISMA Checklist. This file outlines each item on the 2020 PRISMA Checklist and notes where it can be found in this manuscript.

**Additional file 4.** Components of direct medical costs. This file illustrates the categories of direct medical costs which comprise the total direct medical costs, including room charges, consumables, diagnostics, medications, consults, and services.

## Data Availability

All data relevant to the study are included in the article or uploaded as additional files. Additional template data extraction forms are available from the corresponding author upon reasonable request.
