# Supplementary material for: Cost of childhood RSV management and cost-effectiveness of RSV interventions: a systematic review from a low- and middle-income country perspective
Source: BMC Med. 2023 Mar 31;21:121. doi: 10.1186/s12916-023-02792-z (PMC10067246; doi:10.1186/s12916-023-02792-z)
Supplement: Supplementary file 1 — Additional file 1. Search keywords. This file lists all search terms that were used to search Embase, MEDLINE, and Global Index Medicus via the Embase search interface. The three components of the search terms were groups of terms related to each topic: “RSV”, “Economics”, and “Low and middle income countries”. [file 12916_2023_2792_MOESM1_ESM.docx]

## Additional File 1: Search keywords

The following terms were used to search Embase, MEDLINE, and Global Index Medicus via the Embase search interface.

| **Search Component** | **Keywords used** |
| --- | --- |
| RSV | 'human respiratory syncytial virus'/exp OR 'human respiratory syncytial virus' OR 'respiratory syncytial virus infection'/exp OR 'respiratory syncytial virus infection' OR 'respiratory syncytial virus vaccine'/exp OR 'respiratory syncytial virus vaccine' OR 'rsv' |
| Economics | 'cost effectiveness analysis'/exp OR 'cost effectiveness analysis' OR 'cost benefit analysis'/exp OR 'cost benefit analysis' OR 'cost' OR 'cost'/exp OR cost OR 'cost utility analysis'/exp OR 'cost utility analysis' OR 'cost consequence analysis'/exp OR 'cost consequence analysis' OR 'quality adjusted life year'/exp OR 'quality adjusted life year' OR 'disability-adjusted life year'/exp OR 'disability-adjusted life year' OR 'health economics'/exp OR 'health economics' OR 'economic aspect'/exp OR 'economic aspect' OR 'economic impact' OR 'economic burden' OR 'cost of illness' OR 'budget impact analysis' OR 'budget impact model' |
| Low and middle-income countries | 'low income country'/exp OR 'low income country' OR 'middle income country'/exp OR 'middle income country' OR 'gavi' OR 'lmic' OR 'low* income' OR 'low and middle income' OR  'afghanistan' OR 'afghanistan'/exp OR afghanistan OR 'guinea bissau'/exp OR 'guinea bissau' OR 'somalia' OR 'somalia'/exp OR somalia OR 'burkina faso'/exp OR 'burkina faso' OR 'north korea'/exp OR 'north korea' OR 'south sudan'/exp OR 'south sudan' OR 'burundi' OR 'burundi'/exp OR burundi OR 'liberia' OR 'liberia'/exp OR liberia OR 'sudan' OR 'sudan'/exp OR sudan OR 'central african republic'/exp OR 'central african republic' OR 'madagascar' OR 'madagascar'/exp OR madagascar OR 'syrian arab republic'/exp OR 'syrian arab republic' OR 'chad' OR 'chad'/exp OR chad OR 'malawi' OR 'malawi'/exp OR malawi OR 'togo' OR 'togo'/exp OR togo OR 'democratic republic congo'/exp OR 'democratic republic congo' OR 'mali' OR 'mali'/exp OR mali OR 'uganda' OR 'uganda'/exp OR uganda OR 'eritrea' OR 'eritrea'/exp OR eritrea OR 'mozambique' OR 'mozambique'/exp OR mozambique OR 'yemen' OR 'yemen'/exp OR yemen OR 'ethiopia' OR 'ethiopia'/exp OR ethiopia OR 'niger' OR 'niger'/exp OR niger OR 'gambia' OR 'gambia'/exp OR gambia OR 'rwanda' OR 'rwanda'/exp OR rwanda OR 'guinea' OR 'guinea'/exp OR guinea OR 'sierra leone'/exp OR 'sierra leone' OR 'angola' OR 'angola'/exp OR angola OR 'honduras' OR 'honduras'/exp OR honduras OR 'philippines' OR 'philippines'/exp OR philippines OR 'algeria' OR 'algeria'/exp OR algeria OR 'india' OR 'india'/exp OR india OR 'samoa' OR 'samoa'/exp OR samoa OR 'bangladesh' OR 'bangladesh'/exp OR bangladesh OR 'indonesia' OR 'indonesia'/exp OR indonesia OR 'sao tome and principe'/exp OR 'sao tome and principe' OR 'belize' OR 'belize'/exp OR belize OR 'iran' OR 'iran'/exp OR iran OR 'senegal' OR 'senegal'/exp OR senegal OR 'benin' OR 'benin'/exp OR benin OR 'kenya' OR 'kenya'/exp OR kenya OR 'solomon islands'/exp OR 'solomon islands' OR 'bhutan' OR 'bhutan'/exp OR bhutan OR 'kiribati' OR 'kiribati'/exp OR kiribati OR 'sri lanka'/exp OR 'sri lanka' OR 'bolivia' OR 'bolivia'/exp OR bolivia OR 'kyrgyzstan' OR 'kyrgyzstan'/exp OR kyrgyzstan OR 'tanzania' OR 'tanzania'/exp OR tanzania OR 'cape verde'/exp OR 'cape verde' OR 'laos' OR 'laos'/exp OR laos OR 'tajikistan' OR 'tajikistan'/exp OR tajikistan OR 'cambodia' OR 'cambodia'/exp OR cambodia OR 'lesotho' OR 'lesotho'/exp OR lesotho OR 'timor leste'/exp OR 'timor leste' OR 'cameroon' OR 'cameroon'/exp OR cameroon OR 'mauritania' OR 'mauritania'/exp OR mauritania OR 'tunisia' OR 'tunisia'/exp OR tunisia OR 'comoros' OR 'comoros'/exp OR comoros OR 'federated states of micronesia'/exp OR 'federated states of micronesia' OR 'ukraine' OR 'ukraine'/exp OR ukraine OR 'congo' OR 'congo'/exp OR congo OR 'mongolia' OR 'mongolia'/exp OR mongolia OR 'uzbekistan' OR 'uzbekistan'/exp OR uzbekistan OR 'cote d`ivoire'/exp OR 'cote d`ivoire' OR 'vanuatu' OR 'vanuatu'/exp OR vanuatu OR 'morocco' OR 'morocco'/exp OR morocco OR 'djibouti' OR 'djibouti'/exp OR djibouti OR 'myanmar' OR 'myanmar'/exp OR myanmar OR 'egypt' OR 'egypt'/exp OR egypt OR 'nepal' OR 'nepal'/exp OR nepal OR 'gaza strip palestine'/exp OR 'gaza strip palestine' OR 'el salvador'/exp OR 'el salvador' OR 'nicaragua' OR 'nicaragua'/exp OR nicaragua OR 'zambia' OR 'zambia'/exp OR zambia OR 'eswatini' OR 'eswatini'/exp OR eswatini OR 'nigeria' OR 'nigeria'/exp OR nigeria OR 'zimbabwe' OR 'zimbabwe'/exp OR zimbabwe OR 'ghana' OR 'ghana'/exp OR ghana OR 'pakistan' OR 'pakistan'/exp OR pakistan OR 'haiti' OR 'haiti'/exp OR haiti OR 'papua new guinea'/exp OR 'papua new guinea' OR 'albania' OR 'albania'/exp OR albania OR 'american samoa'/exp OR 'american samoa' OR 'argentina' OR 'argentina'/exp OR argentina OR 'armenia' OR 'armenia'/exp OR armenia OR 'azerbaijan' OR 'azerbaijan'/exp OR azerbaijan OR 'belarus' OR 'belarus'/exp OR belarus OR 'bosnia and herzegovina'/exp OR 'bosnia and herzegovina' OR 'botswana' OR 'botswana'/exp OR botswana OR 'brazil' OR 'brazil'/exp OR brazil OR 'bulgaria' OR 'bulgaria'/exp OR bulgaria OR 'china' OR 'china'/exp OR china OR 'colombia' OR 'colombia'/exp OR colombia OR 'costa rica'/exp OR 'costa rica' OR 'cuba' OR 'cuba'/exp OR cuba OR 'dominica' OR 'dominica'/exp OR dominica OR 'dominican republic'/exp OR 'dominican republic' OR 'equatorial guinea'/exp OR 'equatorial guinea' OR 'ecuador' OR 'ecuador'/exp OR ecuador OR 'fiji' OR 'fiji'/exp OR fiji OR 'gabon' OR 'gabon'/exp OR gabon OR 'georgia (republic)'/exp OR 'georgia (republic)' OR 'grenada' OR 'grenada'/exp OR grenada OR 'guatemala' OR 'guatemala'/exp OR guatemala OR 'guyana' OR 'guyana'/exp OR guyana OR 'iraq' OR 'iraq'/exp OR iraq OR 'jamaica' OR 'jamaica'/exp OR jamaica OR 'jordan' OR 'jordan'/exp OR jordan OR 'kazakhstan' OR 'kazakhstan'/exp OR kazakhstan OR 'kosovo' OR 'kosovo'/exp OR kosovo OR 'lebanon' OR 'lebanon'/exp OR lebanon OR 'libyan arab jamahiriya'/exp OR 'libyan arab jamahiriya' OR 'malaysia' OR 'malaysia'/exp OR malaysia OR 'maldives' OR 'maldives'/exp OR maldives OR 'marshall islands'/exp OR 'marshall islands' OR 'mauritius' OR 'mauritius'/exp OR mauritius OR 'mexico' OR 'mexico'/exp OR mexico OR 'moldova' OR 'moldova'/exp OR moldova OR 'montenegro (republic)'/exp OR 'montenegro (republic)' OR 'namibia' OR 'namibia'/exp OR namibia OR 'republic of north macedonia'/exp OR 'republic of north macedonia' OR 'panama' OR 'panama'/exp OR panama OR 'paraguay' OR 'paraguay'/exp OR paraguay OR 'peru' OR 'peru'/exp OR peru OR 'romania' OR 'romania'/exp OR romania OR 'russian federation'/exp OR 'russian federation' OR 'serbia' OR 'serbia'/exp OR serbia OR 'south africa'/exp OR 'south africa' OR 'saint lucia'/exp OR 'saint lucia' OR 'saint vincent and the grenadines'/exp OR 'saint vincent and the grenadines' OR 'suriname' OR 'suriname'/exp OR suriname OR 'thailand' OR 'thailand'/exp OR thailand OR 'tonga' OR 'tonga'/exp OR tonga OR 'turkey (republic)'/exp OR 'turkey (republic)' OR 'turkmenistan' OR 'turkmenistan'/exp OR turkmenistan OR 'tuvalu' OR 'tuvalu'/exp OR tuvalu |
| Other Limits | Year 2000-Jan 20 2022, humans, English language |
