# Supplementary material for: Cost of childhood RSV management and cost-effectiveness of RSV interventions: a systematic review from a low- and middle-income country perspective
Source: BMC Med. 2023 Mar 31;21:121. doi: 10.1186/s12916-023-02792-z (PMC10067246; doi:10.1186/s12916-023-02792-z)
Supplement: Supplementary file 2 — Additional file 2. Article quality checklists. Table S3.1. This table summarizes the quality score of each evaluated cost-effectiveness analysis article for inclusion, using the Drummond checklist. Table S3.2. This table summarizes the quality score of each evaluated cost-of-illness article for inclusion, using the Drummond checklist. [file 12916_2023_2792_MOESM2_ESM.docx]

## Additional File 2: Article quality checklist

### Table S3.1 Article quality scoring for cost-effectiveness analyses

| **Drummond Checklist Item** | Argentina  *Rodriguez* | Argentina  *Fariña* | China  L*iu* | Global  *Li* | Global  *Baral* | Mali  *Laufer* | Malaysia  *Chan* | Mexico  *Salinas-Escudero* | Turkey  *Öncel* | Turkey  *Pouwels* |
| --- | --- | --- | --- | --- | --- | --- | --- | --- | --- | --- |
| Percent (>50% to pass): | 86% | 79% | 81% | 97% | 100% | 97% | 85% | 100% | 64% | 100% |
| **Study Design** | *--* | *--* |  | *--* | *--* | *--* | *--* | *--* | *--* | *--* |
| The research question is stated. | 1 | 1 | 1 | 1 | 1 | 1 | 1 | 1 | 1 | 1 |
| The economic importance of the research question is stated. | 1 | 1 | 1 | 1 | 1 | 1 | 1 | 1 | 1 | 1 |
| The viewpoint(s) of the analysis are clearly stated and justified | 1 | 1 | 1 | 1 | 1 | 1 | 1 | 1 | 0 | 1 |
| The study population is clearly described | 1 | 1 | 1 | 1 | 1 | 1 | 1 | 1 | 1 | 1 |
| The rationale for choosing or interventions compared is stated | 1 | 1 | 1 | 1 | 1 | 1 | 1 | 1 | 1 | 1 |
| The alternatives being compared are clearly described | 1 | 1 | 1 | 1 | 1 | 1 | 1 | 1 | 1 | 1 |
| The form of economic evaluation used is stated | 1 | 1 | 1 | 1 | 1 | 1 | 1 | 1 | 1 | 1 |
| **Data collection** | *--* | *--* | *--* | *--* | *--* | *--* | -- | *--* | *--* | *--* |
| The source(s) of effectiveness estimates used are stated | 1 | 1 | 1 | 1 | 1 | 1 | 1 | 1 | 1 | 1 |
| Details of design and results of effectiveness study are given | 0 | 0 | 1 | 1 | 1 | 1 | 0 | 1 | 0 | 1 |
| Methods of synthesis or meta-analysis of estimates are given | *NA* | *NA* | *NA* | 1 | 1 | *NA* | *NA* | NA | *NA* | 1 |
| The primary outcome measure(s) are clearly stated | 1 | 1 | 1 | 1 | 1 | 1 | 1 | 1 | 1 | 1 |
| Methods to value benefits are stated | 1 | 1 | 0 | 1 | 1 | 1 | 1 | 1 | 1 | 1 |
| Details of from whom valuations were obtained were given | 1 | 1 | *NA* | 1 | *NA* | *NA* | 1 | *NA* | 1 | 1 |
| Productivity changes (if included) are reported separately. | *NA* | 0 | 0 | *NA* | *NA* | *NA* | *NA* | *NA* | *NA* | 1 |
| The relevance of productivity changes is discussed. | *NA* | 1 | 0 | *NA* | *NA* | *NA* | *NA* | *NA* | *NA* | 1 |
| Quantities of resource use reported separately from unit costs. | 1 | 0 | 0 | 0 | 1 | 1 | 0 | 1 | 0 | 1 |
| Methods for the estimation of quantities and costs described | 1 | 1 | 0 | 1 | 1 | 1 | 1 | 1 | 0 | 1 |
| Currency and price data are recorded. | 1 | 1 | 1 | 1 | 1 | 1 | 1 | 1 | 1 | 1 |
| Details of currency of price adjustments for inflation given | 0 | 0 | 1 | *NA* | 1 | 1 | 1 | 1 | 0 | 1 |
| Details of any model used are given. | 1 | 1 | 1 | 1 | 1 | 1 | 1 | 1 | 1 | 1 |
| The choice of model and key parameters are justified | 1 | 1 | 1 | 1 | 1 | 1 | 1 | 1 | 1 | 1 |
| **Analysis and interpretation of results** | *--* | *--* | *--* | *--* | *--* | *--* | *--* | *--* | *--* | *--* |
| Time horizon of costs and benefits is stated | 1 | 1 | 1 | NA | 1 | 0 | 1 | 1 | 0 | 1 |
| The discount rate(s) is stated | 0 | 0 | 1 | 1 | 1 | 1 | 0 | 1 | 0 | 1 |
| The choice of discount rate(s) is justified | *NA* | 0 | 1 | 1 | 1 | 1 | *NA* | 1 | *NA* | 1 |
| An explanation is given if costs and benefits not discounted. | 0 | *NA* | *NA* | *NA* | *NA* | *NA* | 0 | 1 | *NA* | 1 |
| Details of statistical tests are given for stochastic data | 1 | 1 | 0 | 1 | 1 | 1 | 1 | 1 | 1 | 1 |
| The approach to sensitivity analysis is given | *NA* | 1 | 1 | 1 | 1 | 1 | *NA* | 1 | 0 | 1 |
| The choice of variables for sensitivity analysis is justified. | *NA* | 1 | 1 | 1 | 1 | 1 | *NA* | 1 | *NA* | 1 |
| The ranges over which the variables are varied are justified | *NA* | 1 | 1 | 1 | 1 | 1 | *NA* | 1 | *NA* | 1 |
| Relevant alternatives are compared. | 1 | 1 | 1 | 1 | 1 | 1 | *NA* | 1 | 1 | 1 |
| Incremental analysis is reported. | 1 | 1 | 1 | 1 | 1 | 1 | 1 | 1 | 1 | 1 |
| Major outcomes are presented in a disaggregated form | 1 | 0 | 1 | 1 | 1 | 1 | 1 | 1 | 0 | 1 |
| The answer to the study question is given. | 1 | 1 | 1 | 1 | 1 | 1 | 1 | 1 | 1 | 1 |
| Conclusions follow from the data reported. | 1 | 1 | 1 | 1 | 1 | 1 | 1 | 1 | 0 | 1 |
| Conclusions are accompanied by the appropriate caveats | 1 | 1 | 1 | 1 | 1 | 1 | 1 | 1 | 1 | 1 |

### Table S3.2 Article quality scoring for cost-of-illness analyses

| **Drummond Checklist Item** | Argentina  *Marcone* | Bangladesh  *Bhuiyan* | China  *Liu* | Colombia  *Rodriguez-Martinez* | Colombia  *Buendia* | Jordan  *Khuri-Bulos* | Malawi  *Baral* | Malaysia  *Sam* | Malaysia  *Chan* | Mexico  *Comas-Garcia* | Thailand  *Bhuket* |
| --- | --- | --- | --- | --- | --- | --- | --- | --- | --- | --- | --- |
| Percent (>50% to pass): | 83% | 94% | 83% | 100% | 100% | 33% | 94% | 94% | 85% | 71% | 83% |
| **Study Design** | *--* | *--* | *--* | *--* | *--* | *--* | *--* | *--* | *--* | *--* | *--* |
| The research question is stated. | 1 | 1 | 1 | 1 | 1 | 1 | 1 | 1 | 1 | 1 | 1 |
| The economic importance of the research question is stated. | 1 | 1 | 1 | 1 | 1 | 0 | 1 | 1 | 1 | 1 | 1 |
| The viewpoint(s) of the analysis are clearly stated and justified | 1 | 1 | 1 | 1 | 1 | 0 | 1 | 1 | 1 | 1 | 1 |
| The study population is clearly described | 1 | 1 | 1 | 1 | 1 | 1 | 1 | 1 | 1 | 1 | 1 |
| The rationale for choosing or interventions compared is stated | *NA* | *NA* | *NA* | *NA* | *NA* | *NA* | *NA* | *NA* | 1 | *NA* | *NA* |
| The alternatives being compared are clearly described | *NA* | *NA* | *NA* | *NA* | *NA* | *NA* | *NA* | *NA* | 1 | *NA* | *NA* |
| The form of economic evaluation used is stated | *NA* | *NA* | *NA* | *NA* | *NA* | *NA* | *NA* | *NA* | 1 | *NA* | *NA* |
| **Data collection** | *--* | *--* | *--* | *--* | *--* | *--* | *--* | *--* | -- | *--* | *--* |
| The source(s) of effectiveness estimates used are stated | *NA* | *NA* | *NA* | *NA* | *NA* | *NA* | *NA* | *NA* | 1 | *NA* | *NA* |
| Details of design and results of effectiveness study are given | *NA* | *NA* | *NA* | *NA* | *NA* | *NA* | *NA* | *NA* | 0 | *NA* | *NA* |
| Methods of synthesis or meta-analysis of estimates are given | *NA* | *NA* | *NA* | *NA* | *NA* | *NA* | *NA* | *NA* | *NA* | *NA* | *NA* |
| The primary outcome measure(s) are clearly stated | 1 | 1 | 1 | 1 | 1 | 0 | 1 | 1 | 1 | 1 | 1 |
| Methods to value benefits are stated | *NA* | *NA* | *NA* | *NA* | *NA* | *NA* | *NA* | *NA* | 1 | *NA* | *NA* |
| Details of from whom valuations were obtained were given | *NA* | *NA* | *NA* | *NA* | *NA* | *NA* | *NA* | *NA* | 1 | *NA* | *NA* |
| Productivity changes (if included) are reported separately. | 1 | 1 | 1 | 1 | 1 | 1 | 1 | 1 | *NA* | *NA* | 1 |
| The relevance of productivity changes is discussed. | *NA* | *NA* | *NA* | *NA* | *NA* | *NA* | *NA* | *NA* | *NA* | *NA* | *NA* |
| Quantities of resource use reported separately from unit costs. | 0 | 1 | 0 | 1 | 1 | 0 | 0 | 1 | 0 | 0 | 0 |
| Methods for the estimation of quantities and costs described | 1 | 1 | 1 | 1 | 1 | 0 | 1 | 0 | 1 | 0 | 1 |
| Currency and price data are recorded. | 1 | 1 | 1 | 1 | 1 | 0 | 1 | 1 | 1 | 0 | 0 |
| Details of currency of price adjustments for inflation given | 1 | 1 | 0 | 1 | 1 | 0 | 1 | 1 | 1 | 0 | 0 |
| Details of any model used are given. | 1 | 1 | 1 | 1 | 1 | 0 | 1 | *NA* | 1 | 1 | 1 |
| The choice of model and key parameters are justified | 1 | 1 | 1 | 1 | 1 | 0 | 1 | *NA* | 1 | 1 | 1 |
| **Analysis and interpretation of results** | *--* | *--* | *--* | *--* | *--* | *--* | *--* | *--* | *--* | *--* | *--* |
| Time horizon of costs and benefits is stated | 1 | 1 | 1 | 1 | 1 | 0 | 1 | 1 | 1 | 1 | 1 |
| The discount rate(s) is stated | *NA* | *NA* | *NA* | *NA* | *NA* | *NA* | *NA* | *NA* | 0 | *NA* | *NA* |
| The choice of discount rate(s) is justified | *NA* | *NA* | *NA* | *NA* | *NA* | *NA* | *NA* | *NA* | *NA* | *NA* | *NA* |
| An explanation is given if costs and benefits not discounted. | *NA* | *NA* | *NA* | *NA* | *NA* | *NA* | *NA* | *NA* | 0 | *NA* | *NA* |
| Details of statistical tests are given for stochastic data | 1 | 1 | 1 | 1 | 1 | 0 | 1 | 1 | 1 | 1 | 1 |
| The approach to sensitivity analysis is given | *NA* | *NA* | *NA* | *NA* | *NA* | *NA* | *NA* | *NA* | *NA* | *NA* | *NA* |
| The choice of variables for sensitivity analysis is justified. | *NA* | *NA* | *NA* | *NA* | *NA* | *NA* | *NA* | *NA* | *NA* | *NA* | *NA* |
| The ranges over which the variables are varied are justified | *NA* | *NA* | *NA* | *NA* | *NA* | *NA* | *NA* | *NA* | *NA* | *NA* | *NA* |
| Relevant alternatives are compared. | *NA* | *NA* | *NA* | *NA* | *NA* | *NA* | *NA* | *NA* | *NA* | *NA* | *NA* |
| Incremental analysis is reported. | *NA* | *NA* | *NA* | *NA* | *NA* | *NA* | *NA* | *NA* | 1 | *NA* | *NA* |
| Major outcomes are presented in a disaggregated form | 0 | 1 | 0 | 1 | 1 | 0 | 1 | 1 | 1 | 0 | 1 |
| The answer to the study question is given. | 1 | 1 | 1 | 1 | 1 | 1 | 1 | 1 | 1 | 1 | 1 |
| Conclusions follow from the data reported. | 1 | 1 | 1 | 1 | 1 | 1 | 1 | 1 | 1 | 1 | 1 |
| Conclusions are accompanied by the appropriate caveats | 1 | 1 | 1 | 1 | 1 | 1 | 1 | 1 | 1 | 1 | 1 |
