# Supplementary material for: Cost of childhood RSV management and cost-effectiveness of RSV interventions: a systematic review from a low- and middle-income country perspective
Source: BMC Med. 2023 Mar 31;21:121. doi: 10.1186/s12916-023-02792-z (PMC10067246; doi:10.1186/s12916-023-02792-z)
Supplement: Supplementary file 4 — Additional file 4. Components of direct medical costs. This file illustrates the categories of direct medical costs which comprise the total direct medical costs, including room charges, consumables, diagnostics, medications, consults, and services. [file 12916_2023_2792_MOESM4_ESM.docx]

## Additional File 4: Components of direct medical costs

| Components of direct medical costs per RSV episode |
| --- |
| 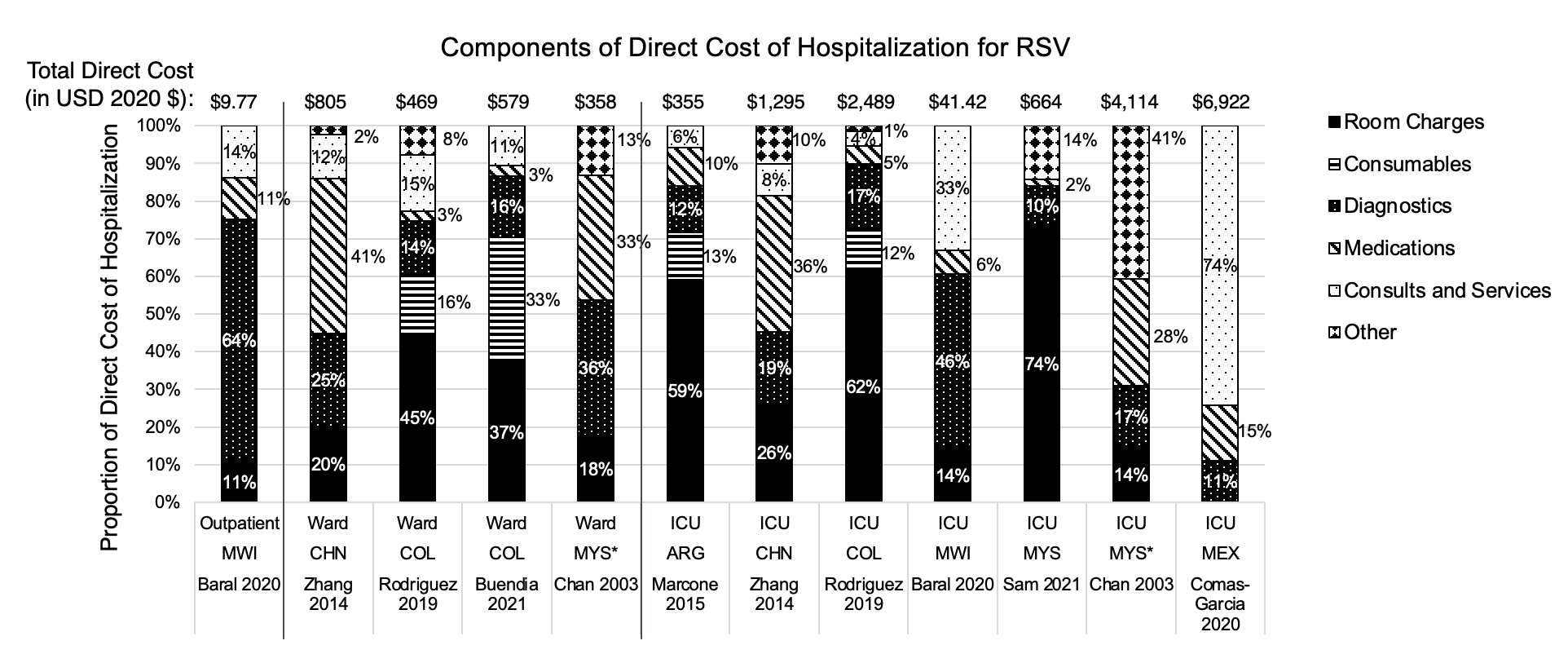 |
| Notes: ^*^ Chan 2003: Comparison groups are for full-term infants vs. pre-term infants, which are included in the Ward and ICU categories, respectively, for comparison by disease severity; Components of direct medical costs were not available for Bhuket et al or Bhuiyan et al.; Currency is reported in 2020 USD |
